# Predicting microRNA targets in time-series microarray experiments via functional data analysis

**DOI:** 10.1186/1471-2105-10-S1-S32

**Published:** 2009-01-30

**Authors:** Brian J Parker, Jiayu Wen

**Affiliations:** 1 The Bioinformatics Centre, Department of Biology, University of Copenhagen, Ole Maaloes Vej 5, 2200 Copenhagen N, Denmark

## Abstract

**Background:**

MicroRNA (miRNA) target prediction is an important component in understanding gene regulation. One approach is computational: searching nucleotide sequences for miRNA complementary base pairing. An alternative approach explored in this paper is the use of gene expression profiles from time-series microarray experiments to aid in miRNA target prediction. This requires distinguishing genuine targets from genes that are secondarily down-regulated as part of the same regulatory module. We use a functional data analytic (FDA) approach, FDA being a subfield of statistics that extends standard multivariate techniques to datasets with predictor and/or response variables that are functional.

**Results:**

In a miR-124 transfection experiment spanning 120 hours, for genes with measurably down-regulated mRNA, exploratory functional data analysis showed differences in expression profiles over time between directly and indirectly down-regulated genes, such as response latency and biphasic response for direct miRNA targets. For prediction, an FDA approach was shown to effectively classify direct miR-124 targets from time-series microarray data (accuracy 88%; AUC 0.96), providing better performance than multivariate approaches.

**Conclusion:**

Exploratory FDA analysis can reveal interesting aspects of dynamic microarray miRNA studies. Predictive FDA models can be applied where computational miRNA target predictors fail or are unreliable, e.g. when there is a lack of evolutionary conservation, and can provide posterior probabilities to provide additional confirmatory evidence to validate candidate miRNA targets computationally predicted using sequence information. This approach would be applicable to the investigation of other miRNAs and suggests that dynamic microarray studies at a higher time resolution could reveal further details on miRNA regulation.

## Background

MicroRNAs (miRNAs) are a class of small non-coding RNAs that are found in both plants and animals. They are known to play important roles in gene regulatory networks by post-transcriptionally regulating the expression of other genes. These miRNAs target other transcripts by forming near-perfect or imperfect base pairings. Such formations silence genes either by mRNA cleavage or translational repression [1].

Computational sequence-based methods have been developed to predict miRNA direct targets. As animal mRNAs have imperfect complementary base pairing to their targets and as they are short in length, most such approaches first search for a perfect 7-mer seed in the 5' end of miRNAs that match to their targets. Searching for such small motifs can lead to high false positive rates and so additional tests such as conservation filters are typically applied [2]. However, although many miRNA targets are conserved in related species, restricting the analysis only to recognizably conserved targets would exclude miRNAs that have recently diverged or changed rapidly. Also, only a small number of such predicted targets have been experimentally validated.

Although animal miRNAs were originally believed to primarily translationally suppress gene expression, they have also been found to lead to mRNA destabilization or degradation. MiRNA transfection microarray experiments capable of detecting such effects at the mRNA level of targets have shown that a large number of transcripts are down-regulated by over-expression of miRNAs [3]. Such high-throughput expression data provide a promising way to assist in miRNA target prediction.

The mRNA expression changes after miRNA transfection could be a result of miRNAs directly targeting these messages (*direct targets *in the sequel), or the mRNA of genes could also be indirectly down-regulated if they are a part of a miRNA-mediated regulatory module controlled by the direct miRNA targets (*indirect targets *in the sequel) [3]. In this case, these indirectly regulated genes' response is causally related to the directly regulated genes in the corresponding module. As such, indirect targets would be expected to respond with delayed expression changes over time relative to the direct targets.

Time-series microarray experiments that repeatedly measure gene expression simultaneously for multiple genes over a time period can capture temporal patterns and dependency of gene expression changes. A recent study by Wang and Wang [4] applied sequence-based target prediction to identify targets and then used a time-series miRNA transfection microarray analysis to experimentally validate the predictions. Another study used a gene set enrichment-like score to measure expression changes at different times [5]. However, these methods treated expression changes over time points as separate observations and therefore did not fully utilize the information on time dependency of gene expression changes. In this paper we aim to explore the differences between the expression profiles of genes that are direct miRNA targets and indirectly targeted genes, including classification of genes based on their profile. We use functional data analysis (FDA) to utilize the higher level structure due to the data being a set of finite samples from a continuous and presumably smooth curve.

Functional data analysis [6-9] is a subfield of statistics applicable when the predictor or response variable can be treated as a set of samples from a function, rather than simply as a feature vector. It is, in essence, the extension of standard multivariate analysis of finite dimensional vector spaces to infinite dimensional function spaces (e.g. reproducing kernel Hilbert spaces (RKHS)) using a functional analytic approach. FDA entails interpolating and smoothing the set of function samples using some appropriate set of basis functions (e.g. splines), kernel smoothing, or regularization. Multivariate procedures such as principal component, regression and classification analyses typically involve an inner product (or some form of metric) defined on the vector space: for the reconstructed continuous function, the conventional multivariate dot product, $\langle x,y\rangle =\sum_{i=1}^{n}x_{i}y_{i}$, can be replaced by the corresponding functional inner product, $\langle x,y\rangle =\int_{0}^{T}x(t)y(t)dt$, for time interval *T*, in the corresponding multivariate analysis method. For example, standard PCA can be extended to functional PCA (FPCA) [6,10] as follows. Conventional multivariate PCA finds the axes of maximum variance in the data by solving the eigenequation *Vξ *= *λξ *for variance – covariance matrix *V *= *n*^-1 ^*X'X*, where *X *is the (mean centred) *n *× *p *data matrix, with *n *samples and *p *features; and *λ *and *ξ *are the corresponding eigenvalue and eigenvector. In functional PCA, the equivalent eigenequation is generalized to *Vξ *= *λξ *where *ξ *is now an eigenfunction and *V *is the covariance operator defined by: *Vx*(*s*) = ∫*v*(*s*, *t*)*x*(*t*)*dt *= ⟨(*v*(*s*, ·), *x*⟩; where the variance-covariance function (the functional extension of the variance-covariance matrix) $v(s,t)=n^{-1}\sum_{i=1}^{n}x_{i}(s)x_{i}(t)$. So, in summary, FPCA can be expressed as the eigenanalysis of the covariance operator *V*, defined by using the covariance function *v *as the kernel of an integral transform, and this formulation of the eigenequation in terms of inner products, ⟨*v*(*s*, ·), *ξ*⟩ = *λξ*, can be applied to either multivariate or functional data, with the respective definition of inner product, where *s *∈ *T *for function domain *T *for the functional case, and index set *T *= 1,..., *p *in the multivariate case. In most cases, this expression can be computed quickly using only matrix expressions utilizing the sampled data points and a matrix of inner products between basis functions, avoiding estimation of the integral [10]. Similarly, most other standard multivariate data analyses such as canonical correlation, cluster, regression and classification analysis can be extended to functional versions.

In this paper, we use FPCA for initial exploratory analysis and a nonparametric functional data analysis (NPFDA) approach [11] for prediction. The advantage of this functional data analytic approach is that the prior knowledge that the data is generated by a continuous smooth dynamics allows for: accurate estimates of derivatives which can then be used in the analysis; effective noise reduction through curve smoothing; applicability to data with irregular time sampling schedules. Such functional data analytic approaches have been previously applied to the analysis of temporal microarray expression data e.g. to cluster time-series gene expression data [12] and to classify yeast cell-cycle gene expression profiles and Dictyostelium cell-type [13]. In this paper, we analyze miRNA-124 transfection time-series microarray expression data using FDA and show that such expression change differences can be modeled as different time activity curves to effectively predict miRNA direct targets and distinguish them from indirect targets.

## Methods

**Figure 1:**
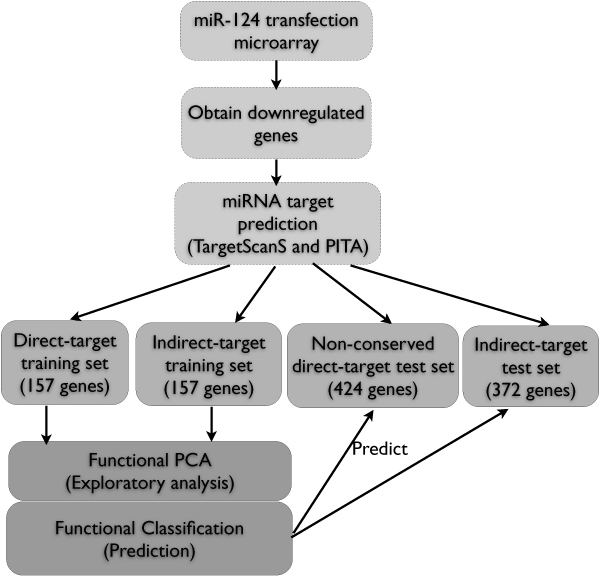
Data analysis flowchart.

We analyzed the miR-124 transfection time-series microarray data previously published in [4], which measures expression levels over 120 hours at 7 time points. We initially identified a set of genes down-regulated after miRNA transfection. To identify, using sequence information alone, a set of true (with high probability) targets and non-targets to train the functional model, we predicted miRNA targets using publicly available target predictors and used the high confidence predictions to determine direct-target and indirect-target sets as training sets for classification. FPCA was used for exploratory data analysis and NPFDA was used to distinguish the direct targets from the indirect targets. An overview of the analysis pipeline in this study is outlined in figure 1 and details are described in the following sections.

### miRNA transfection dataset

We retrieved the miRNA-124 transfection time-series microarray expression dataset from the GEO database [14] (accession no. GDS2657). The dataset contains mRNA expression profiles generated by over-expressing miRNA-124 duplexes into human HepG2 cells in comparison to negative controls at times 4, 8, 16, 24, 32, 72 and 120 hours, using Affymetrix microarrays (U133Plus2; RMA-normalized). miRNA-124 is known to be highly expressed in several tissues including brain and kidney, but is not highly expressed in the cell line used [3,4]. Starting with all genes provided on the U133Plus2 microarray, we first excluded those genes showing low overall expression levels in the control samples as these genes would be unlikely to be able to clearly show down-regulation after miRNA transfection. Only probes where the expression levels over at least half of all time points of the control samples were greater than the median expression level of control samples were retained for further analysis. Expression values were averaged for those genes with corresponding multiple probes.

We next identified genes showing substantial evidence for down-regulation of mRNAs. Genes with over 1.4-fold under-expression (corresponding to a log_2 _expression fold change (FC) of less than -0.5) for at least one time point were considered to be down-regulated genes. We subsequently restricted our analysis to these down-regulated genes in this study (from this down-regulated set, we also excluded a further 19 very noisy genes which had some time points showing greater than 1.3-fold over-expression).

### miRNA target prediction

We obtained two pre-computed target gene sets for miR-124 that were predicted by TargetScanS [15] and PITA [16]. The TargetScanS pre-computed target set for miR-124 contains 289 down-regulated genes with 7- and 8-mer sites conserved across several mammalian species and 384 genes with non-conserved sites. The PITA pre-computed target set for miR-124 contains 198 down-regulated genes with 7- and 8-mer conserved sites and 236 genes with non-conserved sites (a conservation score of higher and lower than 0.9 was considered as the conserved and nonconserved targets, respectively [16]). To construct a control set of genes that were not predicted to be miRNA-124 targets, we further excluded genes showing even weak sites. To do so, we first extracted annotated 3'UTR sequences of genes from Ensemble [17] using Biomart [18]. miRNA target predictions were then performed on the 3'UTRs using PITA [19] with a relaxed parameter setting: a seed of length 6 to 8 bps and allowing G::U pairing in 7- and 8-mer seeds.

### Construction of datasets

We constructed two datasets to be used as training data for functional classification analysis.

(1) *Direct-target training set*: we used the existing computational miRNA target predictors described above to construct a relatively high confidence direct-target training set. As current computational target predictors can vary in their effectiveness [20], to increase confidence we required both predictors to agree. We obtained 157 down-regulated genes that were predicted by both predictors with 7- and 8-mer seed sites and that were found to be evolutionarily conserved in several other species by both predictors.

(2) *Indirect-target training set*: we selected the down-regulated genes that had annotated 3'UTRs but in which no target sites could be found by either predictor and even no weak sites could be found by using PITA prediction with relaxed parameters. From these genes, a set of size identical to the direct-target training set was randomly sampled to form the indirect-target training set.

We also constructed two independent datasets that had not been used for training that we used to further evaluate our model.

(1) *Non-conserved direct-target test set*: we combined the down-regulated genes that were predicted from either of the predictors, yet that had no evidence of conservation. This set consisted of 424 genes and presumably would be enriched for direct targets.

(2) *Indirect-target test set*: we constructed another independent dataset which met the same criteria as the indirect-target training set but excluding genes that had been used for training the model. This set consisted of 372 genes.

### Functional data analysis

We used FPCA to perform exploratory data analysis. To fit a smooth function to the discrete sampled data, we used a set of 9 B-spline basis functions of order 4 (for cubic smoothing splines). Knots were located at the data points. Additional regularized smoothing (*λ *= 0.01) was applied (second derivative roughness penalty).

The NPFDA approach was used for classification. The first derivative of the expression profile was effectively used as the predictor variable, with a boolean (two-class) response variable. The NPFDA used B-spline basis functions (with 7 knots) to produce smooth first derivative estimates. Performance evaluation was by 10 × stratified 10-fold cross-validation (CV). Major parameters were determined via nested CV separately for each fold; other parameter settings were set to their defaults or as appropriate for the data size. To evaluate the discriminability of the classes, the Receiver Operating Characteristics (ROC) curves were calculated as well as the associated area under the ROC curve (AUC).

R code implementing the analysis is available from the authors.

## Results and discussion

### Exploratory data analysis with functional principal components analysis (FPCA)

**Figure 2:**
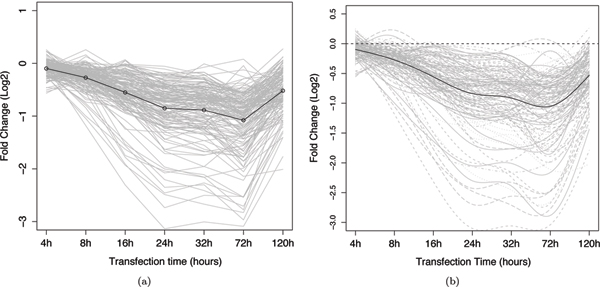
**Profiles of gene expression of the direct-target training set**. Each grey line represents the expression change (y-axis) of one gene across the 7 time points (x-axis). The black line represents the mean value of the curves. (a) plots the linearly interpolated observed data, and (b) plots the corresponding functional data smoothed with B-spline basis functions.

**Figure 3:**
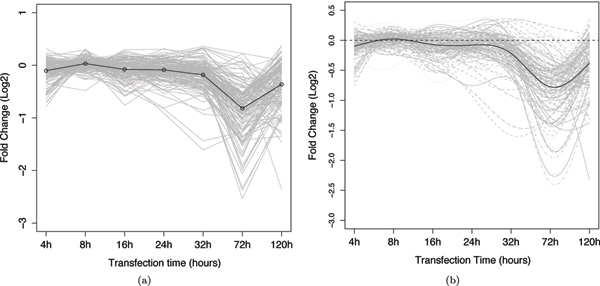
**Profiles of gene expression of the indirect-target training set**. Each grey line represents the expression change (y-axis) of one gene across the 7 time points (x-axis). The black line represents the mean value of the curves. (a) plots the linearly interpolated observed data, and (b) plots the corresponding functional data smoothed with B-spline basis functions.

In the miR-124 dataset, Wang and Wang [4] previously noted that genes directly targeted by miR-124 tended to have substantial down-regulation at time points earlier than the indirectly down-regulated genes. This phenomena is as would be expected as the indirect targets would be causally downstream to the directly regulated genes. Figures 2(a) and 3(a) show the mean, unsmoothed, gene expression profiles of the direct-target training set and the indirect-target training set, respectively (See Methods for construction of the direct-target training and the indirect-target training set). The measurement time points are plotted equally spaced on the time axis to emphasize the early stages of the expression profile. Comparing the mean curves of the two datasets, the direct targets show an immediate decrease in mRNA levels reaching a minimum at approximately 72 hours. By contrast, the indirect targets show a delayed response and do not, on average, commence decreasing until approximately 32 hours, reaching a minimum at about 72 hours. Figures 2(b) and 3(b) show the smoothed functional profiles that were reconstructed using B-spline basis functions (see Methods). As discussed in the background section, it can be seen that smoothed functional curves allow us to reduce noise and have a better estimate of curve derivatives.

**Figure 4:**
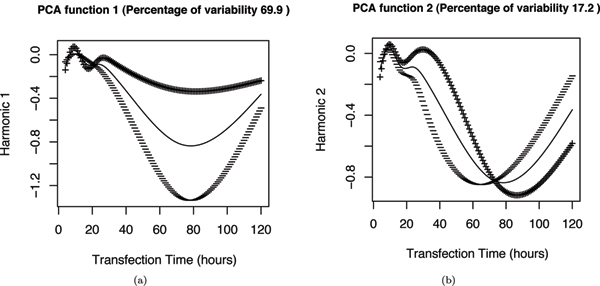
**Functional PCA of the indirect-target training set**. (a) and (b) plot the first two principal component harmonics for the indirect-targets. The thin curve represents the mean fold change and the thick curve represents the effect of adding (+) and subtracting (-) the corresponding FPCA harmonic to the mean curve.

FPCA analysis reveals the modes of variation across the samples. Figure 4 shows the results of FPCA applied to the indirect-target training set and the major modes of variance of the signal compared with the mean (the times shown are linear along the x-axis in these figures). In figure 4(a) the first principal component (PC) function (or harmonic) is added and subtracted from the mean curve, and similarly for the second PC function in figure 4(b). Figure 4(a) shows that there is a large variance in the overall expression fold change along the curves. The curves are normalized in the following classification step to minimize this variance (see below). Figure 4(b) shows that the down-regulation peak varies somewhat in the exact time of its minimum across genes. The extent to which this variance is biologically meaningful and represents causal relationships, as opposed to being explained by the relatively low resolution sampling interval used, will require higher resolution studies to investigate. For example, only approximately three time points were available in this dataset to capture the details of the major indirect target down-regulation peak, which is only just sufficient. Ideally, for an FDA analysis of such noisy data a sampling rate 2 or more times higher than this would be needed to capture fine details well [8].

**Figure 5:**
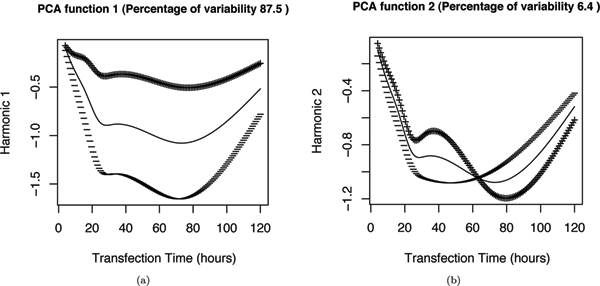
**Functional PCA for the direct-target training set**. (a) and (b) plot the first two principal component harmonics for the direct-targets. The thin curve represents the mean fold change and the thick curve represents the effect of adding (+) and subtracting (-) the corresponding FPCA harmonic to the mean curve.

**Figure 6:**
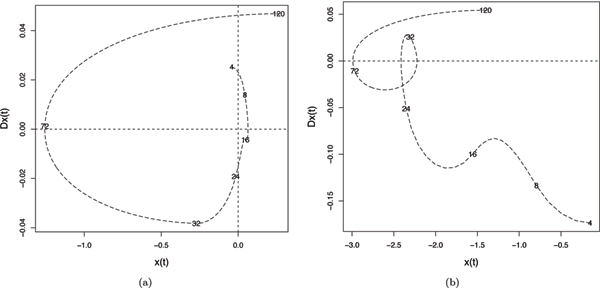
**Phase plane plot for direct and indirect targets**. First derivative, Dx(t), versus expression function, x(t), plot of a representative curve from the (a) direct-target set and (b) indirect target set. The time points are labelled along the curve.

Figure 5 shows the FPCA results for the direct-target training set. Again, a large variance in overall scale in the first channel (figure 5(a)) is seen. Figure 5(b) shows a substantial variance in the time of the major peak at around 72 hours as in the indirect target. However, it also shows evidence of a smaller secondary mode of variance at approximately 24 hours; this would suggest that there is a mixture of components to the curve, with the main direct miRNA repression occurring in the first phase, and a later component matching the response of the indirect targets. This biphasic response is also demonstrated in figure 6 which shows phase plane plots of curve slope against curve value for two representative genes of the direct and indirect target sets. Abrupt changes in such plots (cusps) highlight sudden changes in curve shape. The direct target plot, figure 6(a), shows a cusp at approximately 32 hours, compared with the smooth plot of the indirect target, figure 6(b). Existing kinetic models of miRNA gene regulation have not modeled this effect [21].

### Classification using nonparametric FDA (NPFDA)

As noted above, the gene expression curves show a large variance in overall fold change in the first principal component of the functional PCA. A key preprocessing step in FDA is registration of the curves to remove unimportant amplitude or other variations. Prior to classification, therefore, each curve was standardized to have a mean (log_2_) fold change of -1. Thus, average fold change differences between the direct and indirect target sets were normalized away, and the shape of the response curves alone was effectively used to distinguish the direct targets from the indirect targets. Using curve shape information only would be expected to provide a more robust predictor than also relying on the absolute fold change as a feature, as it varies substantially between genes. An NPFDA discrimination model was trained on the direct and indirect target training sets (see Methods). The performance was compared with linear discriminant analysis (LDA) as an example of a standard multivariate analysis.

**Figure 7:**
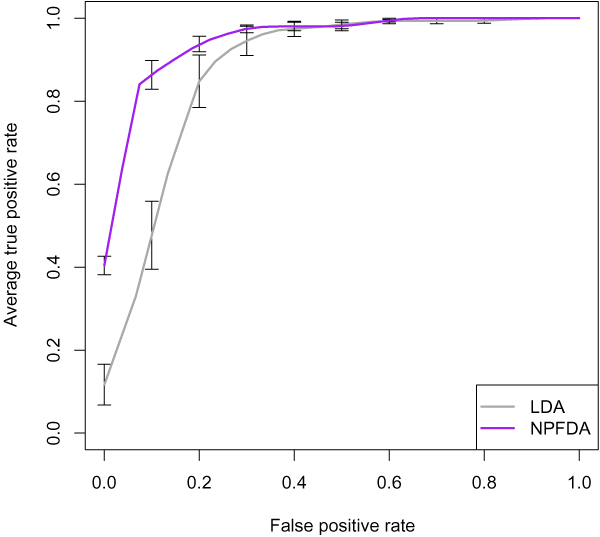
**ROC curve for classification**. The ROC curves plot the true positive (sensitivity) versus false positive (1-specificity) for NPFDA and LDA classification. Error bars show standard deviation over the CV replications.

The results are shown in Table 1. The NPFDA gave very good prediction accuracy of 88% and AUC of 0.96. (AUC is a measure of the discriminative power of the classes using the given features and classifier, and varies from 0.5 for non-distinguishable classes to 1.0 for perfectly distinguishable classes. The AUC can be interpreted as the probability that two random samples from the two classes will be ranked correctly, and is invariant to changes in class proportions (unlike accuracy)). The increase in accuracy over LDA was statistically significant (*p <*0.01; McNemar test). Figure 7 shows the ROC curves for NPFDA and standard LDA: NPFDA shows a substantial improvement over multivariate LDA. For comparison, the discriminability of each time point treated separately as a univariate feature was examined: the expression level across genes at each time point was used directly for ranking to compute the corresponding AUC. The best discriminating time point was at 24 hours with an AUC of 0.91.

**Table 1:** Classification performance using NPFDA and LDA

	Accuracy (standard error)	AUC (standard error)
NPFDA	88% (0.019)	0.96 (0.012)

LDA	83% (0.022)	0.88 (0.020)

**Figure 8:**
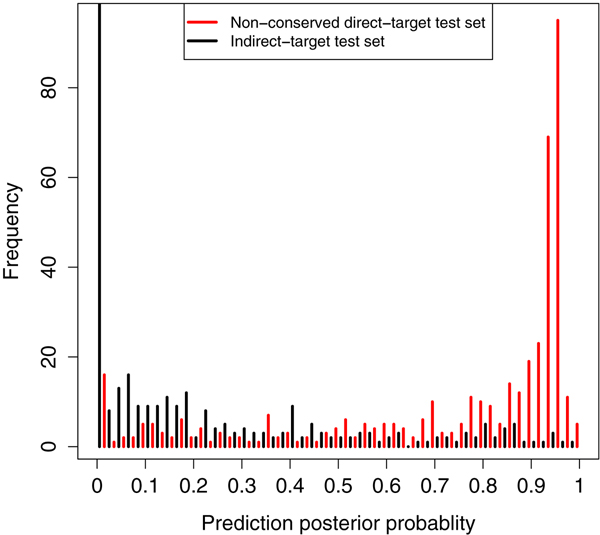
**Distributions of posterior probabilities**. Distributions of the predicted posterior probabilities for two independent sets: nonconserved direct-target test set (red) and indirect-target test set (black).

To further validate the trained model, we also used it to classify data that was independent of the training set: the non-conserved direct-target test set and the indirect-target test set (See Method). Although the true status of these data are not known and so explicit accuracy and AUC results cannot be computed, we would expect the majority of the non-conserved test set to be true direct targets. However without conservation information we would expect a proportion of false positives i.e. indirect targets in actuality. For the indirect-target test set, we would expect predominantly true indirect targets. We generated histograms of the posterior probabilities from the NPFDA in these datasets (figure 8). Comparing the histograms of the non-conserved direct target test set (red) and the indirect-target test set (black), we see this expected result: the red set has overall high posterior probability (for being a direct target) with a large mode approaching 1. Based on this evidence, the majority of the predicted genes without conservation information are indeed true direct targets.

**Figure 9:**
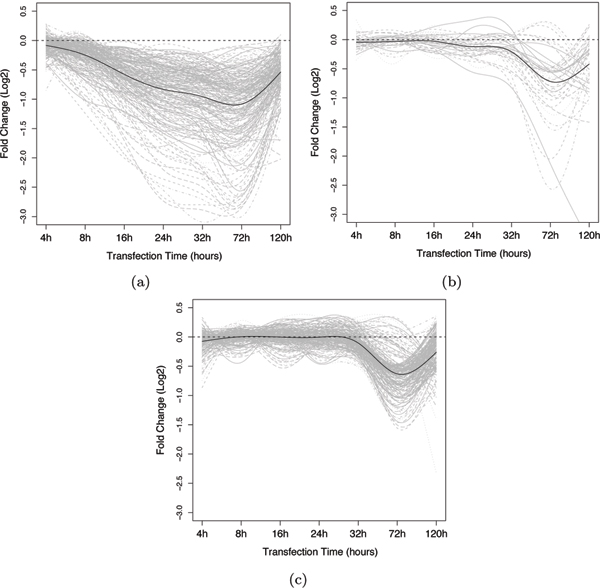
**Profiles of gene expression for predicted classes**. (a) Top nonconserved direct-target test set predictions (posterior probability greater than 80%). (b) Bottom nonconserved direct-target test set predictions (posterior probability less than 20%). (c) Top indirect-target test set predictions (posterior probability less than 20%).

Figure 9(a) shows the time series plots for the top positive predictions of the non-conserved direct target test set (red set in the figure 8) with a posterior probability greater than 0.8 (size 262). These true positives show a profile similar to the direct targets in the training set, which gives high confidence that they are indeed true positives. Taking those high confidence predictions with posterior probability *> *0.9 gives a set of 203 new non-conserved miRNA target predictions (cf. 76 conserved targets predicted in [4]).

Figure 9(b) shows the bottom predictions of the non-conserved direct target test set with a posterior probability of less than 0.2 (size 44). These curves appear to match the indirect target profile seen in the training set indicating that they are likely false positives of the computational predictors. Figure 9(c) shows the time series plots for the top predictions for the indirect-target test set (black set in figure 8) with a posterior probability of less than 0.2 (size 262). These curves match the expected indirect target expression change profile. It also appears that there are at least two subgroups amongst these indirect targets, one with a much longer response time.

## Conclusion

In this study, we presented an FDA analysis of the differences in expression profiles between direct and indirect miRNA targets in a miR-124 miRNA transfection experiment. An exploratory FDA analysis showed differences in response latency, with direct targets showing an immediate down-regulation whereas indirect targets typically showed an approximately 32 hour delay. Also, direct miRNA target curves show evidence of a biphasic, two-component response with an initial early decrease presumably due to direct effects of the miRNA on mRNA, and a later component matching the response of the indirect targets, possibly due to secondary effects.

These time profile differences can be utilized for classification, and in the prediction analysis we showed that FDA can provide very good discrimination, substantially better than a standard multivariate analysis, as FDA utilizes the prior knowledge that the biological process of regulation generates a smoothly varying time profile. Such a predictive model would be especially useful in cases where computational approaches are less reliable e.g. lack of evolutionary conservation. Further, this approach can be used to provide additional confirmatory evidence (posterior probabilities) for computationally predicted miRNA targets and so improve computational miRNA target prediction. This approach would be applicable to the investigation of other miRNAs and these results suggest that dynamic microarray studies at a higher time resolution could reveal further details on miRNA regulation.

## Competing interests

The authors declare that they have no competing interests.

## Authors' contributions

BJP and JW designed the study and analyzed the data. Both authors drafted, read and approved the final manuscript. BJP and JW contributed equally to this work.
